# Ultrasound clusters of joint inflammation in systemic lupus erythematosus: a cross-sectional study

**DOI:** 10.1007/s00296-025-05986-1

**Published:** 2025-09-24

**Authors:** Francesco Natalucci, Fulvia Ceccarelli, Claudia Ciancarella, Simona Truglia, Francesca Romana Spinelli, Cristiano Alessandri, Fabrizio Conti

**Affiliations:** Lupus Clinic, Reumatologia, Dipartimento di Scienze Cliniche, Anestesiologiche Internistiche e Cardiovascolari, Sapienza Università di Roma, Viale del Policlinico, 155, Rome, 00161 Italy

**Keywords:** Systemic lupus erythematosus, Joint involvement, Arthritis, Ultrasound, Cluster analysis

## Abstract

Despite the high frequency of Systemic Lupus Erythematosus (SLE)-related joint involvement, few studies investigated the anatomical distribution of this manifestation. In the present study, by applying musculo-skeletal ultrasound (US), we aimed at mapping joint involvement in a large cohort of SLE patients. We enrolled SLE patients with past or present joint involvement. US evaluation was performed at level of metacarpophalangeal joints (MCPs), proximal interphalangeal (PIPs) joints, wrists, knees, and metatarsophalangeal (MTPs) joints. Grey scale synovitis and Power Doppler were scored according to the EULAR-OMERACT ultrasound scoring system (range 0–3). Principal Component Analysis (PCA) and Unsupervised Hierarchical Cluster Analysis (CA) were performed to define the presence of clusters. As control we evaluated patients with RA. We evaluated 119 SLE patients and consequently 4046 joints. US synovitis ≥ 1 was detected in 375 joints (9.3%). The wrist was the most commonly involved joint [right (R): 63.5%, left (L) 54.2%]. Ninety-six joints (2.3%) had at least grade 2 synovitis, mainly at wrists (R 8.40%, L 7.56%) and knees (R 6.72%, L 5.8%). PCA identified four clusters: medium-large joints (wrists and knees), MTPs; PIPs and MCPs. This result was confirmed by applying the correlation matrix. Finally, a similar clusters distribution was observed when using CA. Our analysis demonstrated as wrists and knees were the most commonly involved joints according to US assessment. Through a multi-statistical approach, we demonstrated the presence of at least three different US-detected clusters.

**Supplementary Information:**

The online version contains supplementary material available at 10.1007/s00296-025-05986-1.

## Introduction

Systemic Lupus Erythematous (SLE) is an autoimmune disease characterized by a great clinical heterogeneity, with different severity degrees [1, 2]. Among the disease- related manifestations, joint involvement is extremely common and it can be identified in up to 90% of patients during disease course [3]. It could vary from inflammatory arthralgia to severe polyarthritis resembling Rheumatoid Arthritis (RA) [4, 5]. The presence of this manifestation significantly impacts on the patient’s quality of life and affects daily life activities [6]. From a clinical perspective, joint involvement in SLE patients has been well-defined: it is generally characterized by the symmetric involvement of hands small joints, while large joints were less frequently involved [7]. In the last decades several studies tried to deeper characterize joint involvement in SLE patients by using imaging techniques, as previously done for RA. In particular, US assessment has been largely applied to evaluate SLE-related joint involvement, unmasking the presence of subclinical synovitis that potentially could lead to bone erosion (BE) development [4]. In the present study we aimed at evaluating the anatomical distribution and the severity of SLE-related joint involvement by applying US assessment in a large cohort. Furthermore, we applied a multi-statistical approach to detect the presence of different clusters in the distribution of US-detected synovitis.

## Patients and methods

We performed a cross-sectional study including SLE patients with past and/or present joint involvement attending at the Lupus Clinic of Sapienza University of Rome (*Sapienza Lupus Cohort*). Patients satisfying the 2019 EULAR/ACR criteria were enrolled for the present analysis [8]. Clinical, demographic, and laboratory features of enrolled patients were collected in a standardized computer-filled form.

Each patient received a complete physical evaluation including tender and swollen joint count (TJC and SJC, respectively). Furthermore, the Visual Analogue Scale (VAS) for pain and disease activity was assessed (0–100 mm). Therefore, we evaluated the activity of joint involvement at the time of inclusion in the study by using the Disease Activity Score with 28 joints (DAS28). Finally, we evaluated global disease activity by using SLEDAI-2k [9]. As controls for the US assessment, we enrolled consecutive RA patients attending at the same rheumatologic center.

This study was reported following the STROBE guidelines as recommended by the EQUATOR Network.

The study was approved by the Ethics Committee of the Sapienza University of Rome/ Policlinico Umberto I, Rome, Italy (Protocol number 3270, approval date 09 September 2014). All the patients signed informed consent.

### Ultrasonographic assessment

US was performed by using a My Lab Eight Exp (Esaote, Genova, Italy) machine equipped with a multifrequency linear array transducer (6–18 MHz) with the application of Power Doppler (PD, PFR 750 Hz, Doppler frequency 11.1 MHz, gain 50% and low filters).

US evaluation was performed in the following sites: metacarpophalangeal (MCPs) joints, proximal interphalangeal (PIPs) joints, wrists, knees, and metatarsophalangeal (MTPs) joints. According to the OMERACT definitions, grey scale (GS) synovitis and power Doppler (pD) were scored using a semi-quantitative scale [10, 11]. In detail, the GS synovitis was scored as follows: 0 = no synovial hypertrophy, 1 = mild hypertrophy, 2 = moderate hypertrophy, and 3 = severe hypertrophy. For the pD scoring, the following definition was considered: 0 = absence of signal; 1 = mild hyperemia, one or two vessels signal (including one confluent vessel); 2 = moderate hyperemia, and less than 50% of GS area; 3 = marked hyperemia, vessels signal in more than half of the synovial area.

Furthermore, a systematic bilateral multiplanar grey scale examination was performed at the level of MCPs, PIPs, MTPs, and knees to identify the presence of bone erosions (BE), according to the OMERACT definitions [11]. The US examination was performed by a trained rheumatologist (FC) with 20 years of experience in the field of musculoskeletal US.

### Statistical analysis

Normally distributed variables were summarized using the mean ± standard deviation (SD), and non-normally distributed variables by the median and interquartile range (IQR). Frequencies were expressed by percentage. Wilcoxon’s matched pairs test and the paired *t*-test were performed accordingly. Univariate comparisons between nominal variables were calculated using the chi-square test or Fisher’s exact test, where appropriate. GraphPad 9.0 (La Jolla CA, USA) software was used for the statistical analysis.

### Cluster analysis and principal component analysis

We performed an unsupervised hierarchical Cluster Analysis (CA) to identify the aggregation of patients into different subgroups sharing common US-detected joint involvement. Euclidean distance and the Ward agglomerative method were applied. Each variable was considered as a single cluster and combined with a neighboring variable (determined by the Euclidean distance). A dendrogram was used to show the process of clustering and the distances between the different clusters. We applied principal component analysis (PCA) on US-detected synovitis to assess clusters of joints prone to be concurrently involved in individuals. Statistical analyses were performed by IBM SPSS Statistics for Windows v26.0 (IBM Corp., Armonk, NY) and GraphPad 9.0 (La Jolla CA, USA).

## Results

We included 119 SLE patients (M/F 8/111) with past and/or present joint involvement. At the enrolment, the median age was 50.1 years (IQR 16.7) and the median disease duration was 162 months (IQR 204). Furthermore, as control, we enrolled 135 RA patients (M/F 36/99; median age 58 years, IQR 15.5; median disease duration 96 months, IQR 141.0).

Focusing on SLE patients, the main clinical/laboratory features and ongoing treatments are summarized in Table 1. Considering overall SLE cohort, we registered a median DAS28 equal to 3.67 (IQR 1.9) and a median SLEDAI-2k equal to 2 (IQR 4). At the time of the visit, fifty-three patients (44.5%) had clinically evident arthritis in at least one joint. In this subgroup of patients, we found a median DAS28 equal to 4.35 (IQR 1.61) and SLEDAI-2k equal to 4 (IQR 2.25). In the RA group, we registered a median DAS28 equal to 3.9 (IQR 2.26), without significant difference when compared with SLE patients (p = ns).

**Table 1:** SLE cohort description (*N* = 119)

Clinical and laboratory manifestation during disease course (%)	
Skin involvement	55.26
Serositis	15.79
Kidney involvement	18.42
Haematological involvement	53.95
CNS/PNS involvement	9.21
Thrombotic events	5.26
Low complement level	35.53
anti-dsDNA	55.26
anti-SSA	61.84
anti-SSB	35.53
anti-RNP	47.37
anti-Sm	48.68
LAC	26.32
aCL IgG/IgM	36.84
antiB2GPI IgG/IgM	25.00

Treatment at the time of study enrolment (%)	
Glucocorticoids	78.95
Hydroxychloroquine	75.00
Methotrexate	26.32
Mycophenolate Mofetil	21.05
Cyclosporine A	15.79
Azathioprine	23.68
Belimumab	2.63
Rituximab	3.95
Anti-platelet therapy	36.84
Anticoagulant therapy	14.47

*LAC* Lupus anti-coagulant, *CNS* Central Nervous System, *PNS* Peripheral Nervous System, *anti-dsDNA* anti-double-strand DNA antibodies, *anti-SSA* anti-Sjogren syndrome-related antigen A autoantibodies, *anti-SSB* anti-Sjogren syndrome-related antigen B autoantibodies, *anti-RNP* anti ribonucleoprotein antibodies; *anti-Sm* anti-Smith antigen antibodies

**Fig. 1:**
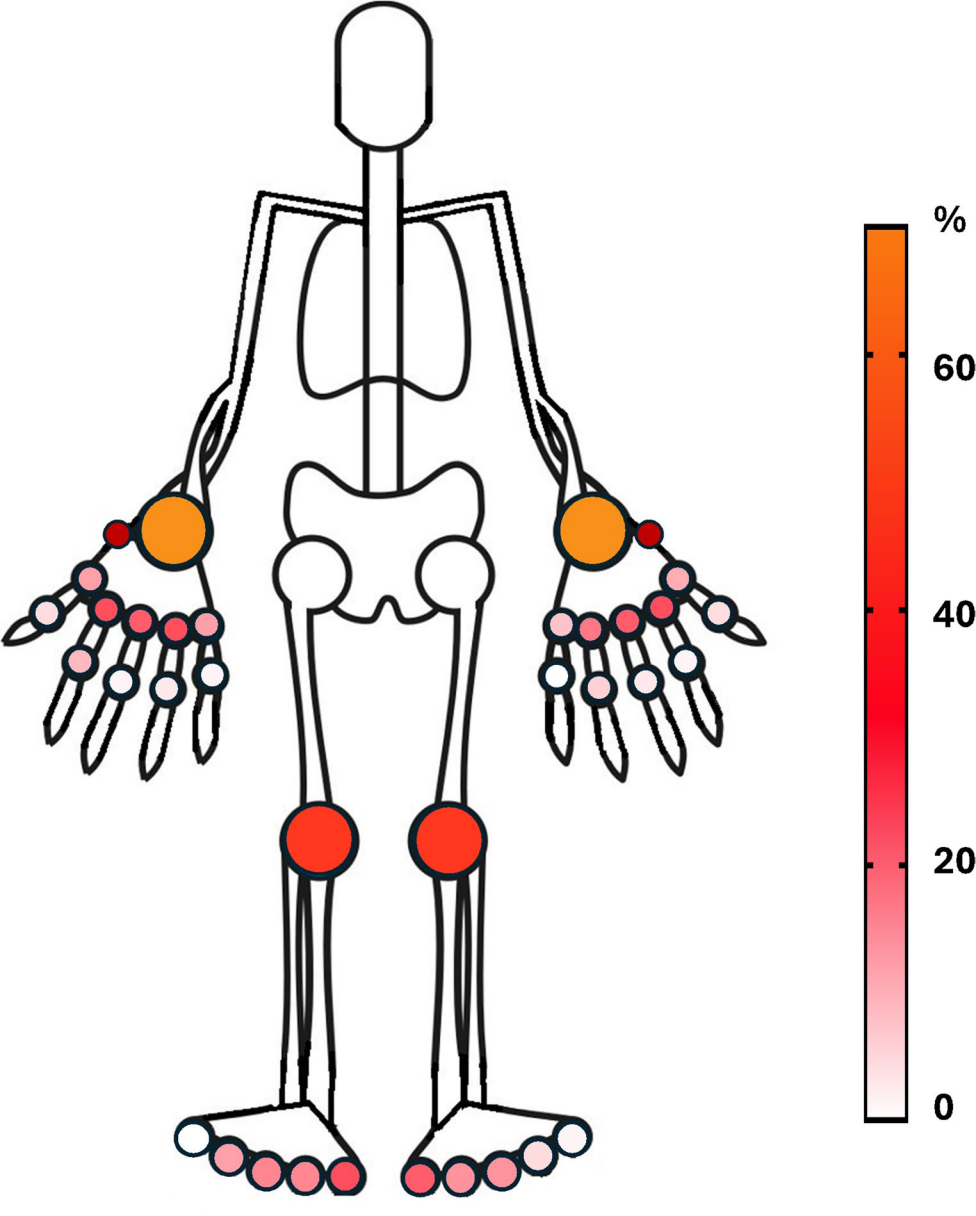
Prevalence of US detected grade ≥ 1 synovitis in systemic lupus erythematosus patients. Percentage refers to each specific joint out of the total analyzed joint. For instance: we detected a Grade **≥** 1 synovitis in R MCP 2 in 14 patients (14/119; 11.7%). A comprehensive report is summarized in Table 2

In the SLE cohort we evaluated a total of 4046 joints. As reported in Tables 2 375 joints (9.3%) showed US synovitis with a grade ≥ 1. The prevalence of synovitis in the different joints was listed in Table 2 and graphically represented in Fig. 1. The radiocarpal joint in the wrist was the most commonly involved, with a prevalence of grade ≥ 1 synovitis equal to 63.5% in the right (R) joint and 54.2% in the left (L) joint, followed by knees (R 20.3%, L 18.6%), 2 MCP joint and 4 MCP joint (R 11.8%, L 9.3% for both joints). Only ninety-six joints (2.3%) had US synovitis with grade ≥ 2, mainly localized at the level of the radiocarpal joint (R 8.4%, L 7.5%) and knees (R 6.7%, L 5.8%). Moving on bone surface evaluation, we observed the presence of BE in 76 joints (1.9%), with a cumulative higher prevalence at level of MCPs joints (48/76, 63.1%).

**Table 2:** Ultrasound features in systemic lupus erythematosus and rheumatoid arthritis patients. The bold text indicates a p value < 0.05

		Systemic Lupus Erythematosus				Rheumatoid Arthritis			Grade 1 synovitis	Grade 2 synovitis	Bone erosions
		Clinically Detected Synovitis	US EULAR-OMERACT Grade 1 Synovitis	US EULAR-OMERACT Grade 2 Synovitis	Bone Erosions	US EULAR-OMERACT Grade 1 Synovitis	US EULAR-OMERACT Grade 2 Synovitis	Bone Erosions	p SLE *versus* RA	p SLE *versus* RA	p SLE *versus* RA
		N (%)	N (%)	N (%)	N (%)	N (%)	N (%)	N (%)			
I MCP	Right	4 (3.39)	8 (6.78)	4 (3.36)	6 (5.04)	14 (10.4)	4 (2.88)	17 (12.6)	0.37	0.82	**0.04**
	Left	2 (1.69)	7 (5.93)	7 (5.88)	6 (5.04)	12 (8.9)	3 (2.16)	17 (12.6)	0.47	0.13	**0.04**
II MCP	Right	14 (11.76)	14 (11.76)	5 (4.20)	12 (10.08)	23 (17.0)	14 (10.07)	41 (30.4)	0.28	0.06	**< 0.0001**
	Left	9 (7.63)	11 (9.32)	3 (2.52)	10 (8.40)	17 (12.6)	7 (5.04)	27 (20)	0.43	0.28	**0.01**
III MCP	Right	12 (10.17)	13 (11.02)	7 (5.88)	2 (1.68)	13 (9.6)	5 (3.60)	10 (7.41)	0.83	0.40	**0.03**
	Left	7 (5.93)	7 (5.93)	2 (1.68)	5 (4.20)	17 (12.6)	6 (4.32)	6 (4.44)	0.08	0.21	0.99
IV MCP	Right	4 (3.39)	14(11.76)	7(5.88)	0 (0.0)	8 (5.9)	2 (1.44)	5 (3.7)	0.11	0.05	0.06
	Left	2 (1.69)	11(9.32)	6 (5.04)	0 (0.0)	12 (8.9)	4 (2.88)	6 (7.41)	0.99	0.38	**0.03**
V MCP	Right	4 (3.39)	8 (6.78)	5 (4.20)	6 (5.04)	8 (5.9)	5 (3.60)	17 (12.6)	0.80	0.82	**0.04**
	Left	2 (1.69)	5 (4.24)	2 (1.68)	7 (5.88)	14 (10.4)	9 (6.47)	18 (13.3)	0.09	0.05	0.05
I PIP	Right	4 (3.39)	3 (2.54)	3 (2.52)	0 (0.0)	4 (2.9)	1 (0.72)	1 (0.74)	0.99	0.25	0.99
	Left	7 (5.93)	3 (2.54)	1 (0.84)	2 (1.68)	5 (3.7)	2 (1.44)	2 (1.48)	0.72	0.48	0.99
II PIP	Right	12 (10.17)	6 (5.08)	1 (0.84)	2 (1.68)	8 (5.9)	1 (0.72)	7 (5.2)	0.99	0.92	0.17
	Left	14 (11.76)	1 (0.85)	0 (0.00)	2 (1.68)	4 (2.9)	2 (1.44)	7 (5.2)	0.37	0.18	0.17
III PIP	Right	14 (11.76)	2 (1.69)	0 (0.00)	0 (0.0)	6 (4.4)	1 (0.72)	6 (4.44)	0.29	0.34	**0.03**
	Left	11 (9.32)	2 (1.69)	0 (0.00)	0 (0.0)	6 (4.4)	2 (1.44)	6 (4.44)	0.29	0.18	**0.03**
IV PIP	Right	4 (3.39)	2 (1.69)	0 (0.00)	0 (0.0)	5 (3.7)	2 (1.44)	3 (2.22)	0.45	0.18	0.24
	Left	3 (2.54)	4 (3.39)	0 (0.00)	0 (0.0)	4 (2.9)	2 (1.44)	4 (2.96)	0.99	0.18	0.12
V PIP	Right	2 (1.69)	1 (0.85)	0 (0.00)	1 (0.84)	2 (1.5)	0 (0.00)	4 (2.96)	0.99	-	0.37
	Left	2 (1.69)	0 (0.00)	0 (0.00)	1 (0.84)	3 (2.2)	1 (0.72)	5 (3.70)	0.25	0.34	0.21
RUC	Right	15 (12.71)	75 (63.56)	10 (8.40)	-	74 (54.8)	38 (27.34)	-	0.16	**< 0.0001**	-
	Left	15 (12.71)	64 (54.24)	9 (7.56)	-	72 (53.3)	44 (31.65)	-	0.90	**< 0.0001**	-
I MTP	Right	0 (0.00)	14 (11.76)	2 (1.68)	1 (0.84)	26 (19.3)	10 (7.19)	4 (2.96)	0.12	**0.03**	0.37
	Left	0 (0.00)	13 (11.02)	2 (1.68)	1 (0.84)	19 (14.0)	7 (5.04)	3 (2.22)	0.57	0.13	0.62
II MTP	Right	0 (0.00)	10 (8.47)	1 (0.84)	1 (0.84)	32 (23.7)	11 (7.91)	2 (1.48)	**0.001**	**0.006**	0.99
	Left	1 (0.85)	9 (7.63)	1 (0.84)	0 (0.0)	28 (20.7)	10 (7.19)	3 (2.22)	**0.004**	**0.01**	0.24
III MTP	Right	0 (0.00)	8 (6.78)	1 (0.84)	0 (0.0)	23 (17.0)	6 (4.32)	0 (0.0)	**0.019**	0.08	-
	Left	0 (0.00)	3 (2.54)	0 (0.00)	1 (0.84)	26 (19.3)	8 (5.76)	1 (0.74)	**0.0001**	**0.008**	0.99
IV MTP	Right	0 (0.00)	7 (5.93)	0 (0.00)	0 (0.0)	14 (10.4)	7 (5.04)	0 (0.0)	0.25	**0.01**	-
	Left	0 (0.00)	3 (2.54)	0 (0.00)	0 (0.0)	11 (8.1)	5 (3.60)	1 (0.74)	0.05	**0.03**	0.99
V MTP	Right	0 (0.00)	0 (0.00)	0 (0.00)	3 (2.52)	3 (2.2)	3 (2.16)	16 (11.8)	0.25	0.10	**0.007**
	Left	0 (0.00)	1 (0.85)	0 (0.00)	3 (2.52)	3 (2.2)	3 (2.16)	16 (11.8)	0.62	0.10	**0.007**
Knee	Right	12 (10.17)	24 (20.34)	8 (6.72)	2 (1.68)	42 (31.1)	24 (17.27)	9 (6.67)	0.06	**0.008**	0.06
	Left	12 (10.17)	22 (18.64)	7 (5.8)	2 (1.68)	33 (24.4)	17 (12.23)	6 (4.44)	0.28	0.07	0.28

*MCP* metacarpophalangeal joint, *PIP* proximal interphalangeal joint, *RUC* radio-ulno-carpal joint (wrist), *MTP* metacarpophalangeal joint, *SLE* systemic lupys erythematosus, *RA* rheumatoid arthritis

**Fig. 2:**
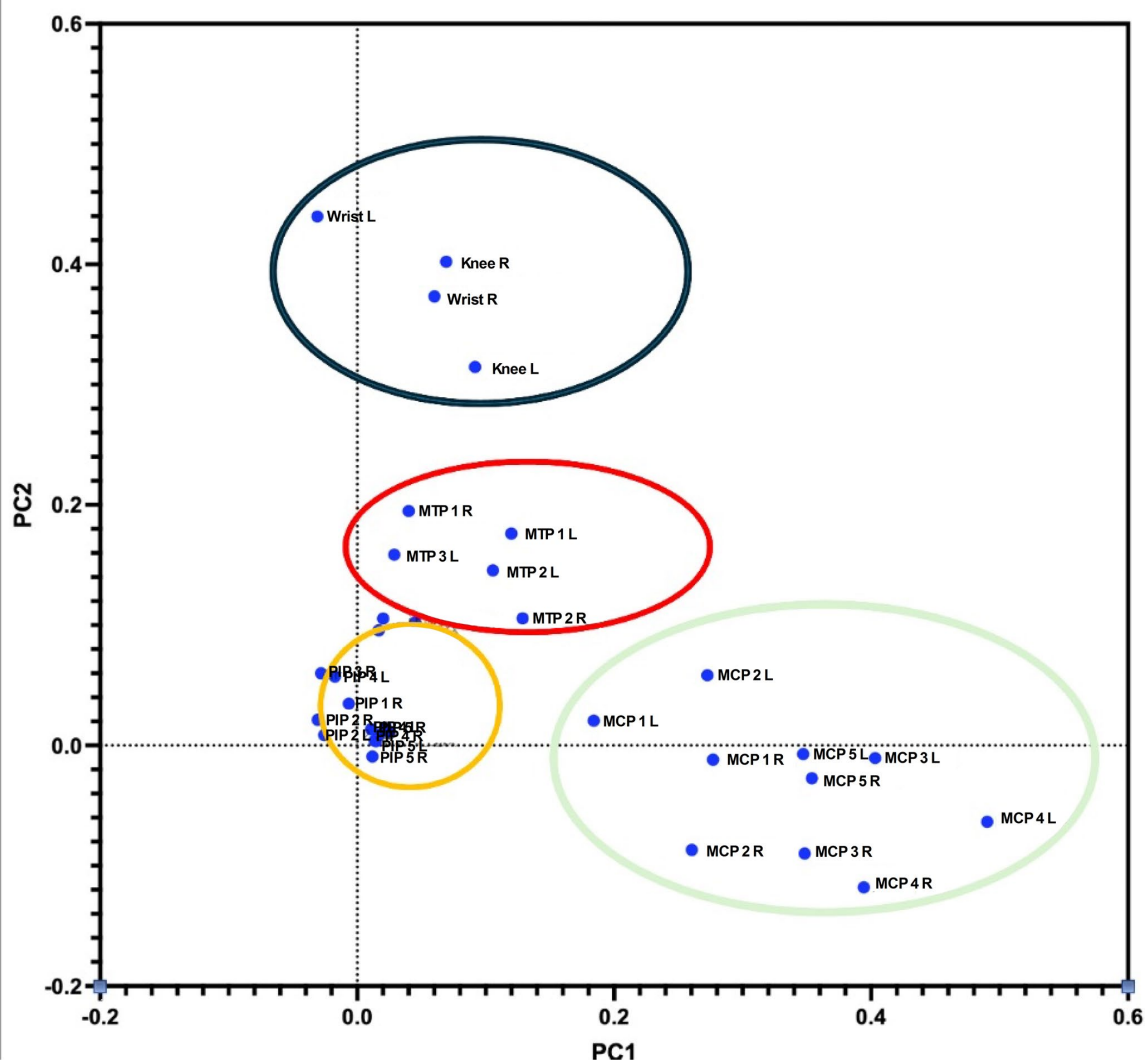
Principal component analysis (PCA) on systemic lupus erythematosus cohort. Principal component analysis on US-detected grade > 1 synovitis. Identified clusters are circled in different colors. Black medium-large joints (wrists and knees); *Red* metatarsophalangeal joints, *Orange* interphalangeal joints, *Green* metacarpophalangeal joints, *MCP* metacarpophalangeal joints, *PIP* Proximal Interphalangeal joints, *MTP* metacarpophalangeal joint, *R* right, *L* left

To analyze the distribution of US-detected synovitis in our cohort, we applied different statistical approach. First of all, we applied PCA on US-detected synovitis to assess joints prone to be concurrently involved in individuals by using visual clusters. Indeed, we found four apparent clusters, graphically represented in Fig. 2. Indeed, these clusters identified different anatomical sites: the first (black) characterized by the involvement of medium-large joints (in particular wrists and knees), a second one (red) involving MTPs joints, the third (orange) involving PIPs joints, and the fourth one (green) involving MCPs joints (Fig. 2).

**Fig. 3:**
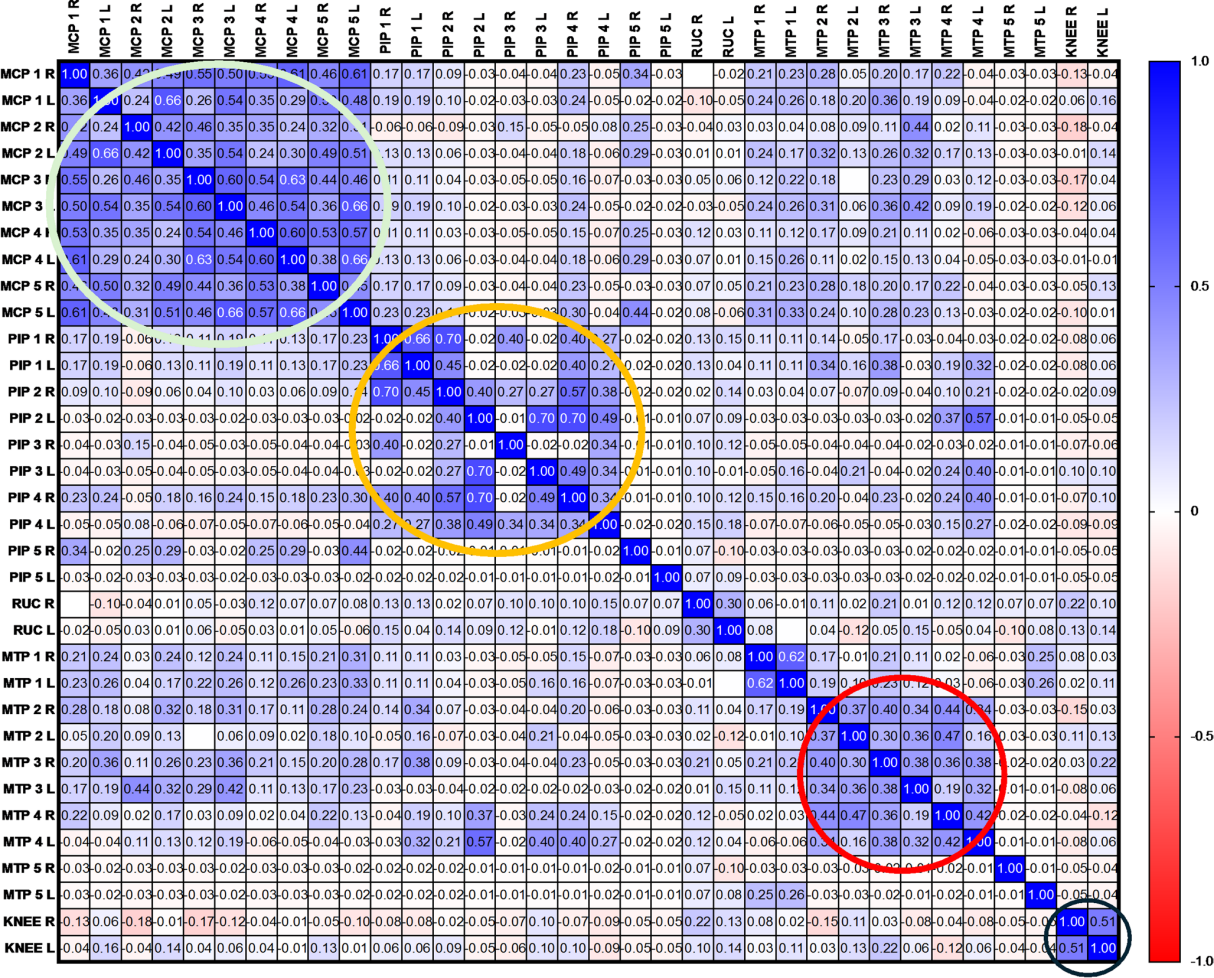
Correlation matrix on systemic lupus erythematosus cohort. Correlation matrix. The values in each cell represent the r Spearman correlation values. Four clusters are identified: Identified clusters are circled in different colors. *Black* joints (knees), *Red* metatarsophalangeal joints, *Orange* interphalangeal joints, *Green* metacarpophalangeal joints

Moreover, we performed a correlation matrix which confirmed the presence of four different clusters as follows: MCPs, PIPs, knees, and wrists (Fig. 3). A further association between MCPs and MTPs joint was detected, with less evidence compared to the other clusters.

**Fig. 4:**
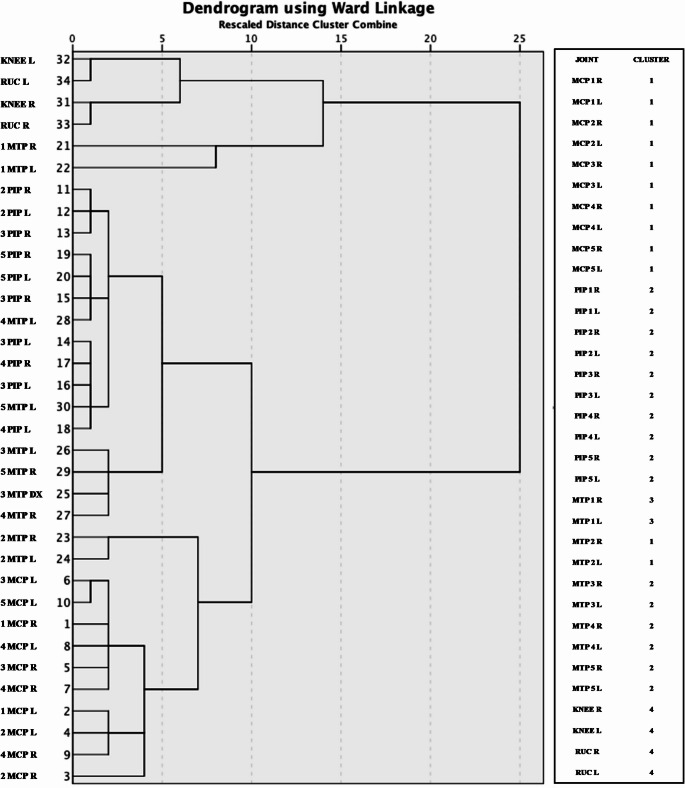
Results from the cluster analysis on systemic lupus erythematosus cohort, represented by dendrogram. Dendrogram representing Cluster Analysis. Euclidean distance and the Ward agglomerative method were applied. *MCP* metacarpophalangeal joints, *PIP* Proximal Interphalangeal joints; *RUC* Radio-Ulno-Carpal joint; *MTP* metacarpophalangeal joint; *R* right, *L* left

We also confirmed these observations with a different approach and statistical method. Indeed, through an unsupervised CA, we identified four different clusters of US synovitis. The involvement of MCPs joints was identified in the cluster 1, PIPs and MTPs were combined into the cluster 2, knee and wrist were aggregated in cluster 4 and finally, 1 MTPs identified a specific cluster (cluster 3). These clusters were represented in Fig. 4.

Finally, we performed a comparison between SLE and RA patients in terms of US-detected synovitis. As expected, RA patients showed a higher number of joints with US-detected synovitis compared with SLE patients (12.8% *versus* 9.3%; *p* < 0.0001). However, when considering each joint separately, a statistical difference was found only for 2 MTP (R: RA 23.7% *versus* SLE 0%, *p* < 0.001; L RA 20.7% *versus* SLE 0.8%, *p* = 0.004) and 3 MTP (R: RA 17% *versus* SLE 0%, *p* = 0.001; L: RA 19.3% *versus* SLE 0%, *p* < 0.0001).

Furthermore, we found a median inflammatory synovitis score significantly higher in RA patients compared to SLE in different joint sites, such as wrists [2 (IQR 1) *versus* 1(IQR 0); *p* < 0.001)], 2 MCP [2 (IQR 1) *versus* 1 (IQR 2); *p* = 0.003] and knees [2 (IQR 1) *versus* 1 (IQR 1); *p* = 0.03)]. All the data were reported in Supplementary Table 1. As expected, the prevalence of bone erosions was significantly higher in RA patients compared to SLE [270/4256 (6.3%) *versus* 76/3776 (3.2%); *p* < 0.0001].

## Discussion

In the present study, we applied US assessment to mapping inflammatory joint involvement in a large SLE cohort. The evaluation of a large SLE cohort and the execution of imaging assessment at different sites demonstrated as US inflammatory changes are prevalently localized in the small-medium joints, with a symmetric distribution and low-grade synovitis. Furthermore, we have been able to identify different US-guided clusters, including respectively medium-large joints, MCPs, and MTPs. At the same time, PIPs joints are usually poorly involved in terms of frequency. Hence, these results confirm the similarities between SLE and RA joint involvement from an imaging point of view in terms of inflammatory changes, but with a more severe erosive damage in RA cohort.

The application of US in a large SLE cohort clearly identified knees and wrists as the most commonly involved joints. The knee involvement in SLE is usually underestimated and the presence of synovitis was only marginally evaluated in previous studies. Only one study was specifically designed to assess knee involvement [12], demonstrating the presence of synovitis in almost half of the evaluated patients. When extrapolating data from other published studies, the prevalence of knee synovitis stands at about 42%, as reported in the literature review published by Zayat and colleagues in 2016 [13]. Furthermore, the same review demonstrated as US inflammatory changes were more common at the level of wrist and MCPs joints, followed by PIPs and MTPs, clearly confirming the additional role of US in characterizing joint involvement [13, 14]. These results are in lines with those observed in our Lupus cohort, where we observed as medium and large joints (in particular, wrists and knees) are more commonly involved by inflammatory changes. Furthermore, when considering low-grade inflammation (grade 1 synovitis) we observed a similar prevalence for SLE and RA cohorts considered in our analysis, while a higher prevalence was observed in RA patients in specific articular site when focusing on grade ≥ 2 synovitis. This could be explained by the higher sensitivity of the US in detecting subclinical inflammation compared with clinical assessment [14–16]. This is a crucial point that reinforces as US is a valuable tool in enhancing clinical evaluation [17, 18]. Our results could suggest the need for a wide US assessment of SLE patients with joint involvement. Furthermore, this evaluation should include a congruent number of joints, including wrists and knees, frequently involved in the inflammatory process. Beyond the prevalence of knees and wrists US-synovitis, we also showed that they tend to be inflamed together, as demonstrated by the association in PCA analysis and CA, suggesting that when one of them is involved, the process may be extended to the other.

PCA and CA, together with the correlation analysis, confirmed the presence of a more classical pattern of SLE joint involvement: in detail, the MCPs involvement could be considered a specific cluster, also showing a close association with the US involvement in MTPs joints. Of note, PCA and CA differentiated these joints into a different cluster from knees and wrists, confirmed by the low association shown in the correlation matrix. This could be explained by the higher prevalence of synovitis in these latter joints, probably masking the real association and splitting them into different groups. According to our results, PIPs joints are not usually involved and when they are, they never reach a moderate inflammation (grade ≥ 2 synovitis), as demonstrated by the PCA and CA analysis. The last group, including the MTPs joints, seems to have intermediate features between PIPs and MCPs. Indeed, they are identified by the PCA and the correlation analysis in a specific group, but at the CA 2 MTP clusters with MCPs and 1 MTP, then identifying a specific different cluster, linked to the absolute higher prevalence of US inflammation, in particular for 1 and 2 MTP.

The comparison between SLE and RA cohorts also gave interesting results: as expected, the absolute number of US-involved joints was significantly higher in RA patients as well as the US inflammatory score. However, when considering each joint separately, a significant difference was demonstrated only for the MTPs and wrists when considering moderate synovitis. This enhances once again the similarities between the joint involvement of these two diseases, also underlying the differences in terms of severity [19, 20]. We provided also information about the US assessment of erosive damage. This evaluation could be interesting in the light of recent evidences demonstrating the presence of erosive damage in SLE-related arthritis, besides the overlap condition of Rhupus [4, 21]. Accordingly, we confirmed the presence of bone erosions in SLE cohort, even though with a significantly lower prevalence compared to RA.

In our opinion, our study has several strengths. Firstly, we systematically evaluated a large cohort of SLE patients assessing 34 joints for each patient, both small and medium-large.

Furthermore, the monocentric design could ensure a homogeneous and consistent ultrasound (US) evaluation, minimizing inter-operator variability, widely considered a common limitation in imaging studies. However, the absence of a pre-defined K parameter could remain a methodological limitation.

Finally, the multi-statistical approach gave us confirmation of the obtained results with different tests, suggesting that what was shown with one test was not driven by casualty. We also acknowledge limitations. The direct comparison between SLE and RA may result in misleading: pathogenesis, clinical manifestation, and management are deeply different, and these factors are surely reflected in US findings. However, both cohorts are composed of consecutively enrolled patients and no differences were found in terms of joint activity, as demonstrated by similar DAS28 values. Moreover, the cross-sectional design limits the power of our findings for two main reasons. First of all, we did not evaluate the progression and trajectory evolution of the cluster over time, and thus the clinical utility of US-clusters. Second, it may have masked the effect of different drugs on the US evaluation. Longitudinal studies are needed to confirm and replicate our findings. The third point concerns the US-assessed joint: we excluded from the analysis ankles, shoulders, hips, and elbows. Generally, these joints were not involved in SLE patients [22], but in the light of our results, a subclinical inflammation can’t be excluded. Moreover, our statistical approach was focused on variable clustering. This did not allow us to define specific groups of patients with peculiar features; however, this is out of the main aim of the study, focused on the identification of US-synovitis clusters. Further studies including these joints are needed to confirm our results.

In conclusion, our study provides novel insights into the ultrasound-based characterization of joint inflammation in SLE patients. To the best of our knowledge this is the first study able to analyze a large SLE cohort by using ultrasound assessment. Another innovative aspect is the joint assessment performed on a large number of joints, thus providing an overall assessment of the inflammatory status of the individual patient. The availability of all these data allowed the application of different statistical modalities, with the possibility to identify different cluster of patients with different phenotypes of joint involvement.

## Supplementary Information

Below is the link to the electronic supplementary material.

Supplementary Material 1

Supplementary Material 2
