# Supplementary material for: Ultrasound clusters of joint inflammation in systemic lupus erythematosus: a cross-sectional study
Source: Rheumatol Int. 2025 Sep 24;45(10):234. doi: 10.1007/s00296-025-05986-1 (PMC12460540; doi:10.1007/s00296-025-05986-1)
Supplement: Supplementary file 2 — Supplementary Material 2 [file 296_2025_5986_MOESM2_ESM.docx]

**Supplementary Table 1.** Inflammatory score comparison between SLE and RA.

|  | **Systemic Lupus Erythematosus** | | **Rheumatoid Arthritis** | |  |
| --- | --- | --- | --- | --- | --- |
|  | **N° Pts** | **Median (IQR)** | **N° Pts** | **Median (IQR)** | **p** |
| **1 MCP** | 15 | 1 (1) | 26 | 1 (1) | 0.39 |
| **2 MCP** | 28 | 1 (2) | 40 | 2 (1) | 0.003 |
| **3 MCP** | 20 | 1 (1) | 32 | 1 (1) | 0.56 |
| **4 MCP** | 25 | 2 (1) | 20 | 1 (1) | 0.12 |
| **5 MCP** | 13 | 2 (1) | 22 | 2 (1) | 0.99 |
| **RUC** | 90 | 1 (0) | 148 | 2 (1) | <0.0001 |
| **1 MTP** | 28 | 1 (0) | 46 | 1 (1) | 0.06 |
| **2 MTP** | 19 | 1 (0) | 57 | 1 (1) | 0.15 |
| **3 MTP** | 11 | 1 (0) | 50 | 1 (1) | 0.49 |
| **4 MTP** | 10 | 1 (0) | 26 | 1 (1) | 0.49 |
| **Knee** | 47 | 1 (1) | 75 | 2 (1) | 0.03 |
